# Impairment of mitochondrial calcium handling in a mtSOD1 cell culture model of motoneuron disease

**DOI:** 10.1186/1471-2202-10-64

**Published:** 2009-06-22

**Authors:** Manoj Kumar Jaiswal, Wolf-Dieter Zech, Miriam Goos, Christine Leutbecher, Alberto Ferri, Annette Zippelius, Maria Teresa Carrì, Roland Nau, Bernhard U Keller

**Affiliations:** 1 Center of Physiology, Georg-August University, Goettingen, Germany; 2 Department of Neurology, Georg-August University, Goettingen, Germany; 3 Laboratory of Neurochemistry, Fondazione S. Lucia IRCCS, Rome, Italy; 4 Department of Biology, University of Rome "Tor Vergata", Rome, Italy; 5 Department of Physics, Georg-August University, Goettingen, Germany; 6 Department of Geriatrics, Evangelisches Krakenhaus, Goettingen-Weende, Germany

## Abstract

**Background:**

Amyotrophic lateral sclerosis (ALS) is a fatal neurodegenerative disorder characterized by the selective loss of motor neurons (MN) in the brain stem and spinal cord. Intracellular disruptions of cytosolic and mitochondrial calcium have been associated with selective MN degeneration, but the underlying mechanisms are not well understood. The present evidence supports a hypothesis that mitochondria are a target of mutant SOD1-mediated toxicity in familial amyotrophic lateral sclerosis (fALS) and intracellular alterations of cytosolic and mitochondrial calcium might aggravate the course of this neurodegenerative disease. In this study, we used a fluorescence charged cool device (CCD) imaging system to separate and simultaneously monitor cytosolic and mitochondrial calcium concentrations in individual cells in an established cellular model of ALS.

**Results:**

To gain insights into the molecular mechanisms of SOD1^G93A ^associated motor neuron disease, we simultaneously monitored cytosolic and mitochondrial calcium concentrations in individual cells. Voltage – dependent cytosolic Ca^2+ ^elevations and mitochondria – controlled calcium release mechanisms were monitored after loading cells with fluorescent dyes fura-2 and rhod-2. Interestingly, comparable voltage-dependent cytosolic Ca^2+ ^elevations in WT (SH-SY5Y^WT^) and G93A (SH-SY5Y^G93A^) expressing cells were observed. In contrast, mitochondrial intracellular Ca^2+ ^release responses evoked by bath application of the mitochondrial toxin FCCP were significantly smaller in G93A expressing cells, suggesting impaired calcium stores. Pharmacological experiments further supported the concept that the presence of G93A severely disrupts mitochondrial Ca^2+ ^regulation.

**Conclusion:**

In this study, by fluorescence measurement of cytosolic calcium and using simultaneous [Ca^2+^]i and [Ca^2+^]_mito _measurements, we are able to separate and simultaneously monitor cytosolic and mitochondrial calcium concentrations in individual cells an established cellular model of ALS. The primary goals of this paper are (1) method development, and (2) screening for deficits in mutant cells on the single cell level. On the technological level, our method promises to serve as a valuable tool to identify mitochondrial and Ca^2+^-related defects during G93A-mediated MN degeneration. In addition, our experiments support a model where a specialized interplay between cytosolic calcium profiles and mitochondrial mechanisms contribute to the selective degeneration of neurons in ALS.

## Background

Amyotrophic lateral sclerosis (ALS) is a fatal neurodegenerative disorder characterized by a selective loss of motor neurons (MNs) in the brain stem, the spinal cord and the motor cortex, leading to progressive weakness, muscle atrophy with eventual paralysis, and death. Approximately 5–10% of ALS cases are familial [1,2]. A decade ago, researchers discovered missense mutations in the gene encoding the Cu/Zn superoxide dismutase 1 (SOD1) in subsets of familial cases; approximately 20% of familial ALS (fALS) is caused by mutations in *SOD1 *with high inter-subject variation of progression, including the point mutation G93A [3-7]

Multiple cascades have been implicated in the motor neuron death pathway, including mitochondrial dysfunction and deformities [8-15]; complex I, III, and IV abnormalities [16-19]; mitochondrial alteration, increase in Ca^2+ ^uptake and increase of cytosolic Ca^2+ ^concentration [6,20], oxidative stress [21]; and glutamate excitotoxicity [22-29]. Alteration and disruption of calcium homeostasis and metabolism [30-40] has also been reported.

The present evidence supports a hypothesis that mitochondrial dysfunction acts with oxidative stress to cause abnormal neurodegeneration via calcium-mediated MN injury. Oxidative stress may lead to increased intracellular calcium, which leads to increased nitric oxide and peroxynitrite formation [21]. Glutamate excitotoxicity may disrupt intracellular calcium homeostasis and reactive oxygen species (ROS) production [27], which may be promoted by oxidative stress as glutamate transporters are particularly susceptible to disruption by oxidants, and oxidative modifications to the transporter have been reported in ALS and the mtSOD1^G93A ^mouse model [24,21]. The etiology is likely to be multifactorial because ALS involves the interplay of several mechanisms to initiate disease and propagate the spread of motor neuron cell death [36,38,39].

Mitochondrial Ca^2+ ^uptake responds dynamically and sensitively to changes in cytosolic Ca^2+ ^levels and plays a crucial role in sequestering the large Ca^2+ ^loads induced by FCCP-evoked Ca^2+ ^influx [41]. The excessive influx of Ca^2+ ^into mitochondria may result in mitochondrial dysfunction. Prominent and sustained mitochondrial depolarization follows intense ion channel receptor stimulation and closely parallels the incidence of neuronal death [42]. Substantial Ca^2+ ^can be accumulated in mitochondria as a result of overloading the matrix with Ca^2+^; this disrupts the structural and functional integrity of the organelle. Hence, mitochondria may be a critical intracellular target of injury after intense Ca^2+ ^channel stimulation and, in this way, may act as a link between massive Ca^2+ ^influx and mitochondria-mediated neurotoxicity by mtSOD1^G93A^. However, the precise relationship between Ca^2+ ^influx, cytosolic Ca^2+ ^increase, and mitochondrial Ca^2+ ^uptake remains obscure.

Culture systems, such as slice culture or primary cultures of MN, have proven to be valuable tools in the physiological and biochemical characterization of ALS-related pathology [43-45]. Still unknown is whether the presence of mtSOD1^G93A ^causes morphological mitochondrial abnormalities when expressed at physiological levels, whether the source specificity of mitochondrial Ca^2+ ^sequestration and spatiotemporal properties of cytosolic Ca^2+ ^([Ca^2+^]i) signaling in cells transfected with wild-type and G93A-mutant SOD1 varies at physiological levels, and if there are functional consequences of changes in mitochondrial function on Ca^2+ ^homeostasis in the presence of mtSOD1^G93A^. To elucidate the underlying molecular events and cellular alterations involved in oxidative stress induced by the aberrant Cu-Zn chemistry and the roles of impaired Ca^2+ ^handling and oxidative stress induced by ROS in fALS, we used SH-SY5Y neuroblastoma cells transfected with the G93A mutant form of *SOD1 *typical for fALS (SH-SY5Y^G93A ^or G93A) and SH-SY5Y cells transfected with wild-type (WT) human *SOD1 *(SH-SY5Y^WT ^or WT) and mimic the situation present in heterozygous patients, previously established as an in-vitro cell culture model of ALS, resembling the situation of heterozygous patients [6,46]. This particular mutation was chosen because it does not affect the activity of SOD1; previously it was shown that SH-SY5Y cells expressing G93A exhibit increased intracellular ROS [47]. The effect of the continuous expression of WT or G93A on mitochondrial morphology and Ca^2+ ^signaling were compared. In addition, the consequences and impact of protonophore FCCP and other organelle-specific drugs on mitochondrial vulnerability and Ca^2+ ^homeostasis were studied.

## Methods

### SH-SY5Y^WT ^and SH-SY5Y^G93A ^transfected neuroblastoma cell cultures

An *in-vitro *model to study the cellular alterations associated with mutations of SOD1 was constructed by transfection of the human neuroblastoma cell line SH-SY5Y with G93A-SOD1 [6]. The cell types that we have used in our study are well characterized and have been used as cellular model system for motoneuron disease by several groups [6,48-50]. The transfected cell lines have relatively low levels of mtSOD/wtSOD and are therefore an attractive model system for the human disease [50]. Upon expression of fALS – SOD1, these cells show several features typical of neurons of both ALS patients and transgenic mice, such as mitochondrial alteration, increased ROS levels and increased cytosolic calcium concentration [see references in [49]].

Transfected human neuroblastoma cell lines constitutively expressing either WT human SOD1 or the G93A mutant form of this enzyme associated with fALS were routinely maintained in Dulbecco's Modified Eagle Medium (DMEM)-F12 (Gibco, Invitrogen, Karlsruhe, Germany) containing 15% fetal calf serum (FCS; Biochrom, Berlin, Germany), 100 U/ml penicillin (Biochrom, Berlin, Germany), and 100 μg/ml streptomycin (Gibco, Invitrogen, Karlsruhe, Germany) at 37°C in a humidified atmosphere with 5% CO_2 _[6]. The cell lines were under consistent selection by addition of 200 μg/ml geneticin (G418 sulfate, Gibco, Invitrogen, Karlsruhe, Germany), which was removed two days before performing the experiments.

### Intracellular fluorometric Ca^2+ ^measurements

The calcium-sensitive fluorescent dye, Fura-2, was introduced into cells by loading the membrane-permeable AM-ester forms (5 μM, 30 min, 37°C, 5% CO_2 _and 95% O_2_), which is termed bolus loading. Fura-2 AM was purchased from Invitrogen (Carlsbad, CA, USA) and freshly dissolved in dimethyl sulfoxide (DMSO) containing 10% pluronic acid before the experiment. Changes in [Ca^2+^]i were measured in non-transfected SH-SY5Y cells (Parental cell line) and SH-SY5Y cells expressing WT or G93A attached to glass cover slips after 2–5 days in culture. Cell layers were incubated with RPMI-1640 (Biochrom, Berlin, Germany) containing 10% FCS (Gibco, Invitrogen, Karlsruhe, Germany) and 0.846 mM Ca^2+ ^(supplier's data). Cells were rinsed with RPMI -1640 and incubated for 20 min at 37°C to allow for complete de-esterification.

Changes in [Ca^2+^]i were monitored by the excitation of Fura-2 AM at 360 nm and 390 nm; emitted light was directed to a dichroic mirror by a beam splitter at 425 nm. In the case of non-transfected SH-SY5Y cells, the excitation of Fura-2 AM was performed at 356 nm and 385 nm and the emitted light was directed to a dichroic mirror with mid-reflection at 425 nm. In some experiments, the light was filtered by a band pass filter of 505–530 nm [See Additional file 1]. Since the background fluorescence is not clearly determinable in AM loaded cells, we regarded the calculated Ca^2+ ^concentrations as an approximation of the real concentration [19]. Therefore, for ester-loaded cells, changes in [Ca^2+^]i are given as changes in the Fura-2 ratio (360/390 nm).

Fura-2 (Kd ~ 0.2 μM) was monitored by a computer-controlled monochromator (Polychrome II, TILL Photonics, Germany), which was connected to an Axioscope microscope (Zeiss, Goettingen, Germany) via quartz fiber optics and a minimum number of optical components for maximum fluorescence excitation (objective Achroplan W 63x, 0.9W). The apparatus was equipped with a CCD camera system [51,52], which displayed 12-bit dynamics (PCO, Germany), and an A/D converter with a 12.5 MHz sampling rate; the binning was set to 4 × 4 and the exposure time was 30–80 ms. Calcium changes in defined regions of interest (ROIs) were monitored online using TILL Vision Software V3.3 (TILL Photonics, Martinsried, Germany). The measured fluorescence ratio at the selected wavelengths was used to calculate the [Ca^2+^]i and was represented as F/F0. Further analysis was performed off-line with the IGOR software (Wavemetrics, Lake Oswego, OR, USA) and Origin software, version 7.5.

### Simultaneous recording of changes in [Ca^2+^]i and [Ca^2+^]mito

Simultaneous fluorometric measurements of [Ca^2+^]i and mitochondrial calcium ([Ca^2+^]mito) were performed utilizing calcium-sensitive fluorescent dyes (Fura-2 AM for cytosolic calcium and Rhod-2 AM for mitochondrial calcium). SH-SY5Y cells expressing either WT or G93A were attached to glass cover slips and loaded initially with 5 μM Fura-2 AM at 37°C for 30 min as described above. Subsequently, cells were washed with RPMI-1640 for 20 min and further incubated with 10 μM Rhod-2 AM for 20 min at 37°C (5% CO_2 _and 95% O_2_). The coverslips were rinsed with RPMI-1640 and further incubated for 20 min at 37°C to allow for complete de-esterification.

To clearly separate and simultaneously monitor the dynamic [Ca^2+^]i and [Ca^2+^]mito profiles with a temporal resolution in the millisecond time domain, we utilized a computer-controlled monochromator that rapidly switched between excitation wavelengths of 390 nm (Fura-2) and 550 nm (Rhod-2) and collected wavelengths above 565 nm for both excitation conditions. Schematic representation of the CCD-imaging setup used for conventional calcium imaging and simultaneous imaging of [Ca^2+^]i and [Ca^2+^]mito signals in the cell line preparation are illustrated in Additional file 1 [See Additional file 1]. For kinetic data analysis, only Fura-2 and Rhod-2 signals from pre-defined regions of cells were used.

The accumulations of mitochondrial and other cell organelles calcium were inhibited by cell organelles specific drug modulators. First, drugs were used, including KCl (K^+^), depolarisation stimulus; FCCP, a protonophore; and oligomycin (oligo), a F_1_, F_0_-ATPase blocker preventing maintenance of the proton gradient, which disrupt the mitochondrial membrane potential (ΔΨ_m_) [53-56]. Secondly, by thapsigargin (thapsi) an extremely tight binding inhibitor of intracellular Ca^2+ ^pumps, which raises cytosolic calcium concentration by blocking the ability of the cell to pump calcium into the sarcoplasmic and endoplasmic reticulum which causes these stores to become depleted [57]. Store-depletion can secondarily activate plasma membrane calcium channels, allowing an influx of calcium into the cytosol. Thirdly, by caffeine, a specific activator of Ca^2+ ^influx from ryanodine receptor (RyR)-dependent Ca^2+ ^stores [58].

### Statistical analysis

Each cover slip was used for a single experiment and included more than 4 cells for each imaging experiment. Unless otherwise indicated, values in the text are given as mean ± standard deviation (SD) and the error bars indicate SD too. All values represent at least three separate experiments. Significance was calculated using the two tailed unpaired Student t-test. A *p*-value < 0.05 was consider statistically significant. Five point smoothing was performed in the case of simultaneous [Ca^2+^]i and [Ca^2+^]mito signals to remove the noise.

### Chemical induction of calcium release

Carbonyl cyanide p-(trifluoromethoxy) phenylhydrazone (FCCP), thapsigargin, and oligomycin were purchased from Sigma-Aldrich Chemie (Deisenhoff, Germany). FCCP was bath applied at a dose of 2 μM. A comparative analysis of the Ca^2+ ^influx in WT and G93A transfected cells was done in the presence of FCCP. For these experiments the SH-SY5Y cells were loaded with 5 μM Fura-2 AM through the incubation method and [Ca^2+^]i transients were triggered once Fura-2 AM concentration was equilibrated after washing. Ca^2+ ^release from intracellular stores by inhibition of the sarcoplasmic/endoplasmic reticulm Ca^2+^-dependent ATPase pump and inhibition of F_1_, F_0_-ATP Synthase was done by intervention of thapsi, and oligo, respectively.

For the investigation of endoplasmic reticulum (ER)-dependent Ca^2+ ^release, caffeine has been established as a specific activator of Ca^2+ ^influx from the RyR-dependent Ca^2+ ^stores [58]. To investigate the relative impact of the ER and mitochondria in [Ca^2+^]i regulation together with the association on MN degeneration in G93A transfected SH-SY5Y cells, we used pharmacological agents as a specific activator of the ER-dependent Ca^2+ ^release response [59], which not only selectively blocks Ca^2+ ^uptake into ER/mitochondrial compartments but also triggers Ca^2+ ^release from intracellular storage sites. Caffeine was purchased from Sigma-Aldrich chemie (Deisenhofen, Germany) and dissolved in water (25 mM). It was then diluted to the final concentration immediately before the experiment. We utilized KCl (K^+^) concentrations of 30 mM for depolarisation induced Ca^2+ ^transients.

## Results

### Impaired mitochondrial responses of cytosolic calcium in G93A transfected cells

Representative non-transfected SH-SY5Y neuroblastoma cells (parental cell line) stained with Fura-2 AM are shown in Additional file 2 [See Additional file 2]. Mitochondrial Ca^2+ ^release responses were measured to verify the impact of mitochondrial uncoupler FCCP in normal SH-SY5Y cells. The application of FCCP for 2 min fairly inhibited mitochondrial Ca^2+ ^and most of the cells had a proper distinguishable and characteristic fluorescence response (See Additional file 2]. In the cells matching set criteria, including a normal morphological appearance of a large soma and intense proper staining, we performed Ca^2+ ^imaging 2–5 days after culture from WT-SOD1 and G93A transfected SH-SY5Y cells.

**Figure 1:**
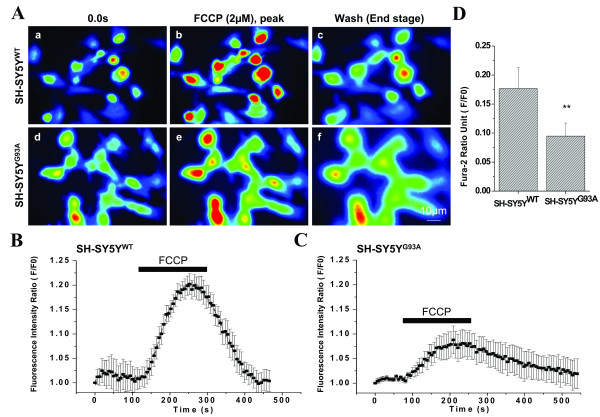
**FCCP induced mitochondrial depolarization in SH-SY5Y neuroblastoma cells transfected with G93A exhibits reduced peak fluorescence amplitude**. A) A CCD imaging photomicrograph series showing [Ca^2+^]i in 7–8 cells before drug application (0.0s), after peak 2 μM FCCP challenge for 3 min, and after FCCP wash in WT (a-c) and G93A (d-f) transfected SH-SY5Y neuroblastoma cells. B) A representative figure of [Ca^2+^]i fluorescence intensity in 6 SH-SY5Y neuroblastoma cells transfected with WT after FCCP application. FCCP (2 μM) induced a fast, transient elevation in [Ca^2+^]i and a fast recovery to baseline. C) In G93A transfected SH-SY5Y neuroblastoma cells, FCCP induced a transient elevation in [Ca^2+^]i fluorescence intensity that was lower in magnitude, followed by a plateau for 1 min, and a delayed recovery to baseline. D) A bar diagram to illustrate the reduction of the sustained Ca^2+ ^response in G93A transfected SH-SY5Y neuroblastoma cells (F/F0 = 0.0948 ± 0.0223; N = 5, n = 23) compared to WT transfected SH-SY5Y cells (F/F0 = 0.1766 ± 0.0362; N = 3, n = 17). Values represent means ± SD, **p < 0.001, scale bar = 10 μm. N = Number of experiments; n = Number of cells.

The transfected cells were treated with FCCP (3 min) and the [Ca^2+^]i measured for both WT and G93A-transfected SH-SY5Y cells (Fig. 1). A CCD imaging photomicrograph series of [Ca^2+^]i in 7–8 cells before drug application (0.0s), after peak FCCP challenge (3 min), and after FCCP wash in WT-SOD1 (a-c) and G93A (d-f) transfected cells are shown in Fig. 1A. Figs. 1B and 1C show [Ca^2+^]i as cytoplasmic calcium versus time curves in 6 representative cells during FCCP challenge. The mitochondrial uncoupler induces a fast transient elevation in [Ca^2+^]i and a fast recovery to baseline in WT transfected cells (Fig. 1B, F/F0 = 0.1766 ± 0.0362; N = 3, n = 17). The G93A transfected cells had a comparatively lower fluorescence intensity for transient elevation in [Ca^2+^]i followed by a delayed recovery to baseline (Fig. 1C, F/F0 = 0.0948 ± 0.0223; N = 5, n = 23). The FCCP-induced calcium influx peak fluorescence intensity in G93A transfected cells was diminished by approximately 48% compared to the calcium influx peak fluorescence intensity of WT transfected cells (Fig. 1D; *p *< 0.001).

Calcium uptake by the mitochondria is accomplished by the mitochondrial calcium uniporter (MCU) located in the inner mitochondrial membrane [60]. The accumulation of mitochondrial Ca^2+ ^may have a perceptible influence on the existence of a [Ca^2+^]i transient. This effect was studied by following the Ca^2+ ^signal under healthy and deleterious mitochondrial conditions in the presence of FCPP. We found that, in G93A transfected SH-SY5Y cells, the Ca^2+ ^influx (peak fluorescence) was diminished compared to WT transfected SH-SY5Y cells, which, peaked within 2–3 min and was followed by a baseline recovery in approximately 2 min. The G93A transfected cells took almost 5–6 min to achieve a baseline recovery. However, the cells transfected with G93A suffered from reduced [Ca^2+^]mito after depletion of mitochondrial Ca^2+ ^stores by FCCP (Fig. 1).

### Effect of high K^+^-evoked Ca^2+ ^transient and its impact on FCCP-induced Ca^2+ ^influx

**Figure 2:**
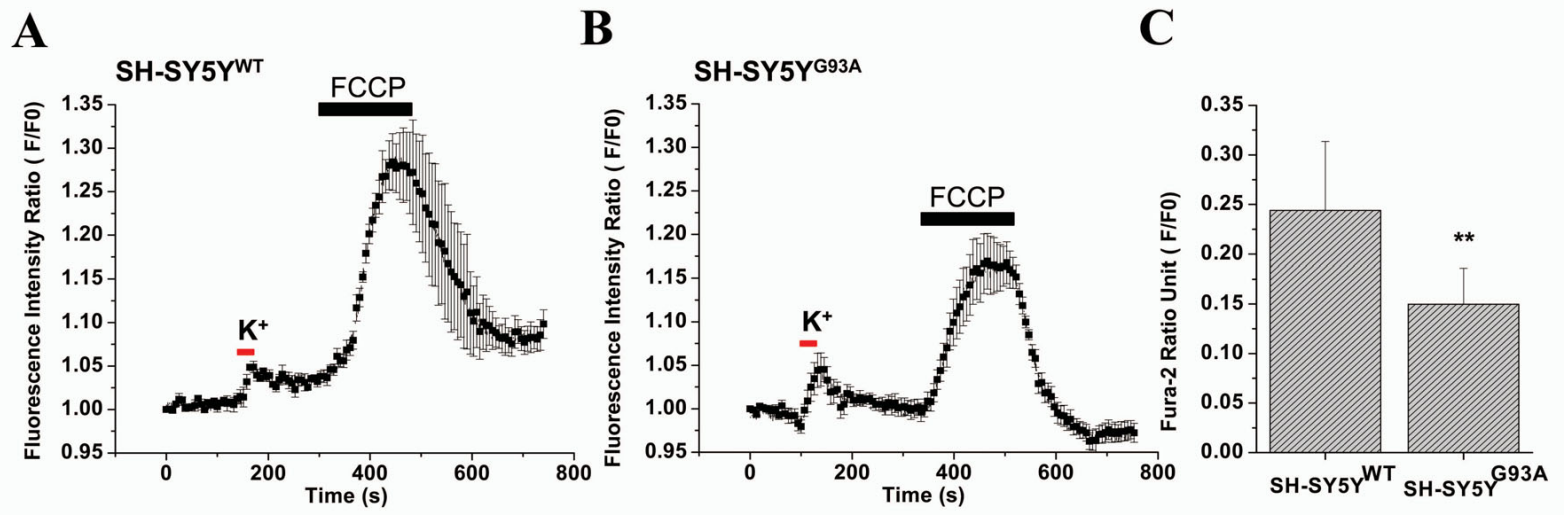
**The effect of high K^+ ^(30 mM) – evoked Ca^2+ ^transient and its impact on the FCCP-induced Ca^2+ ^influx in WT and G93A transfected SH-SY5Y neuroblastoma cells**. A) A representative figure showing effect of perturbing the mitochondrial Ca^2+ ^uptake on the calcium transient evoked by 30 mM K^+ ^depolarizing stimulus for 30s (red horizontal bar) and by 2 μM FCCP-evoked Ca^2+ ^efflux (black horizontal bar). FCCP (2 μM) induced a fast, transient elevation in [Ca^2+^]i and a fast recovery to baseline after depolarization-induced stimulus in 7 SH-SY5Y cells transfected with WT. B) In G93A transfected SH-SY5Y cells, the FCCP-induced [Ca^2+^]i transient elevation after depolarization-induced stimulus was delayed 5–10s and there was a reduction in the magnitude of fluorescence intensity followed by complete recovery to baseline. C) A bar diagram to illustrate the reduction of the sustained Ca^2+ ^response in G93A transfected SH-SY5Y neuroblastoma cells (F/F0 = 0.1497 ± 0.0362; N = 5, n = 20) compared to WT transfected SH-SY5Y cells (F/F0 = 0.2440 ± 0.0696; N = 3, n = 20). Values represent means ± SD, **p < 0.001, scale bar = 20 μm. N = Number of experiments; n = Number of cells.

To analyze the comparative efficiency of mitochondria as a Ca^2+ ^sequestering organelle, FCCP was applied to evacuate mitochondrial Ca^2+^after the WT and G93A transfected cells had been exposed to an evoked Ca^2+ ^load through a depolarizing stimulus of K^+^. First, the SH-SY5Y cells transfected with WT or G93A were stained with Fura-2 AM and exposed to 30 mM K^+ ^for 30 sec, which was followed by a 3 min challenge with FCCP (Fig. 2). As shown in Figs. 2A and 2B, the peak amplitude of FCCP-evoked Ca^2+ ^influx after depolarization was 0.2440 ± 0.0696 (N = 3, n = 20) and 0.1497 ± 0.0362 (N = 5, n = 20) for the WT and G93A transfected cells, respectively. Fig. 2C shows the comparative analysis of average calcium release.

### Interaction between ER/mitochondria in differential Ca^2+ ^store regulation by inhibition of the sarcoplasmic/endoplasmic reticulm Ca^2+^-dependent ATPase pump after pharamacological intervention

The relationships between mitochondrial calcium pools and those discharged by the Ca^2+^-ATPase inhibitor thapsi, an extremely tight-binding inhibitor of intracellular Ca^2+ ^pumps which induces rapid Ca^2+ ^release from intracellular stores by inhibition of the sarcoplasmic/endoplasmic reticulm Ca^2+^-dependent ATPase pump without inositol phosphate formation [57,61] were studied in WT and G93A transfected SH-SY5Y cells to estimate reciprocal functional interplay between the ER and mitochondria. It was previously shown in SOD1^G93A ^mice that release of ER based Ca^2+ ^stores play minimal role and not an essential factors in the death mechanism of ALS vulnerbale MNs [62]. However, contrary to this study it was also shown that Ca^2+ ^release from the ER contributes to neuronal cell death because the Ca^2+^release blocker, dantrolene, can protect neurons against bioenergetic failure and cellular damage [63]. While little is known about the exact ER/mitochondrial Ca^2+ ^regulation mechanism in WT and G93A transfected cells, the restoration of ER function or attenuation of the secondary dysfunction induced by ER could present a new, highly promising mechansim for pharmacological intervention which could bring new ways to treat or minimize neuronal cell injury in the pathological states of ALS.

**Figure 3:**
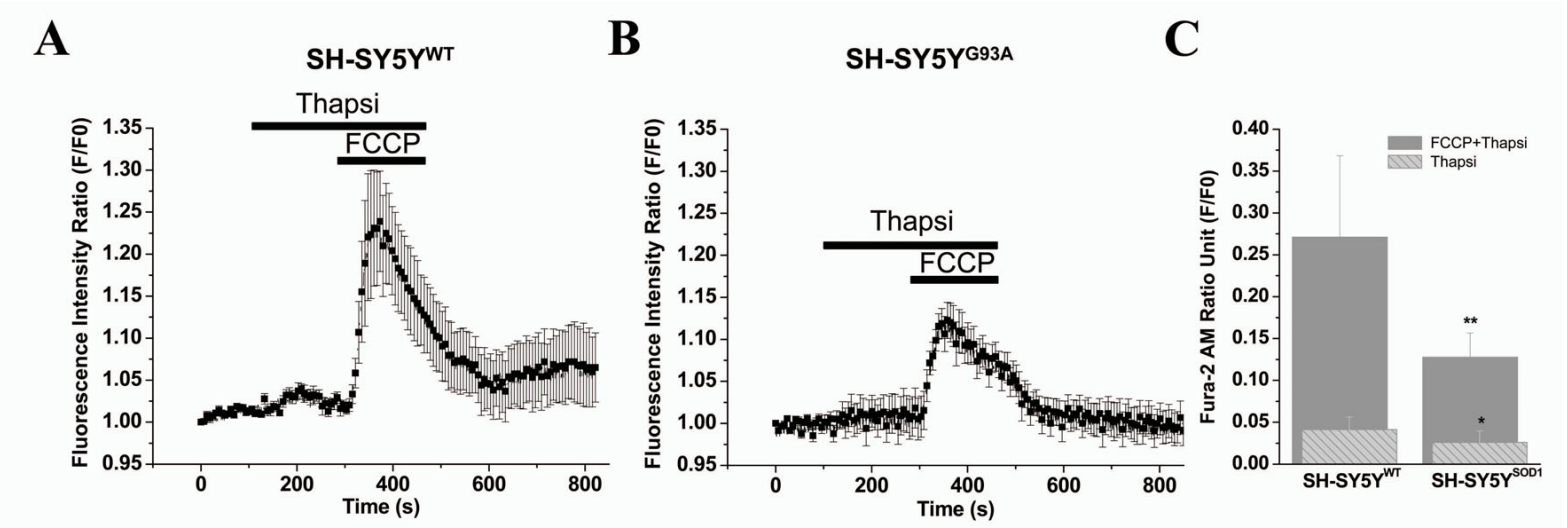
**Analysis of the differential Ca^2+ ^storage and regulation of the ER and mitochondria by pharmacological intervention in WT and G93A transfected SH-SY5Y cells**. Cells were stimulated using thapsi and FCCP, which interfere with the integrity of the ER and mitochondria, respectively, and were used to release Ca^2+ ^from intracellular stores by inhibition of the sarcoplasmic/endoplasmic reticulm Ca^2+^-dependent ATPase pump and mitochondrial stores by protonophore action. A) The quantitative kinetic profile of the thapsi and FCCP-evoked [Ca^2+^]i release in WT transfected SH-SY5Y cells. B) The corresponding quantitative kinetic profile of the thapsi and FCCP-evoked [Ca^2+^]i release in the G93A transfected SH-SY5Y cells. The trace is representative of mean of 4–6 cells in focus stimulated with thapsi (5 μg/ml; 6 min) and 2 μM FCCP (3 min, normalized data). The horizontal black bars indicate the duration of stimulation by thapsi and with FCCP plus thapsi. Fura-2 AM signals are represented as F/F0. C) A bar diagram of thapsi and FCCP plus thapsi-induced Ca^2+ ^release in the WT and G93A transfected SH-SY5Y neuroblastoma cells (N = 3, n = 15). Gray bars represent thapsi plus FCCP-induced Ca^2+ ^release in WT (F/F0 = 0.2712 ± 0.0971) and G93A (F/F0 = 0.1276 ± 0.0287) transfected SH-SY5Y cells. Striped bars represent thapsi-induced Ca^2+ ^release in WT (F/F0 = 0.0412 ± 0.0152) and G93A (F/F0 = 0.0258 ± 0.0137) transfected cells. Values represent means ± SD, *p < 0.01, **p < 0.001. N = Number of experiments; n = Number of cells.

Analysis of the Ca^2+ ^storing ability of ER and mitochondria in WT and G93A transfected cells was done using thapsi and FCCP. We show here that in fura-2-loaded cells, thapsi stimulated a slow plateau phase increase in cytoplasmic Ca^2+ ^concentration in WT and G93A transfected cells. There was a significant quantitative difference between the ER and mitochondrial Ca^2+ ^load in WT and G93A transfected cells; the Ca^2+^release response was high in the mitochondria of WT transfected cells. As shown in Fig. 3, the peak amplitude of mitochondrial Ca^2+ ^release after application of FCCP plus thapsi was 0.2712 ± 0.0971 and 0.1276 ± 0.0287 where as peak amplitude of ER Ca^2+ ^release after application of thapsi was 0.0412 ± 0.0152 and 0.02589 ± 0.0137 in WT and G93A transfected cells, respectively (Figs. 3A–C). The impact of thapsi on the peak amplitude of Ca^2+ ^release was nominal compared with FCCP, indicating a lesser role for ER compared with mitochondria in Ca^2+ ^regulation. Comparative details of the normalized fura-2 ratio in both WT and G93A transfected cells are summarized in Fig. 3C (N = 3, n = 15).

### Interaction of ER/Mitochondria calcium stores regulation by activation of Ca^2+ ^influx from the RyR-dependent Ca^2+ ^stores

**Figure 4:**
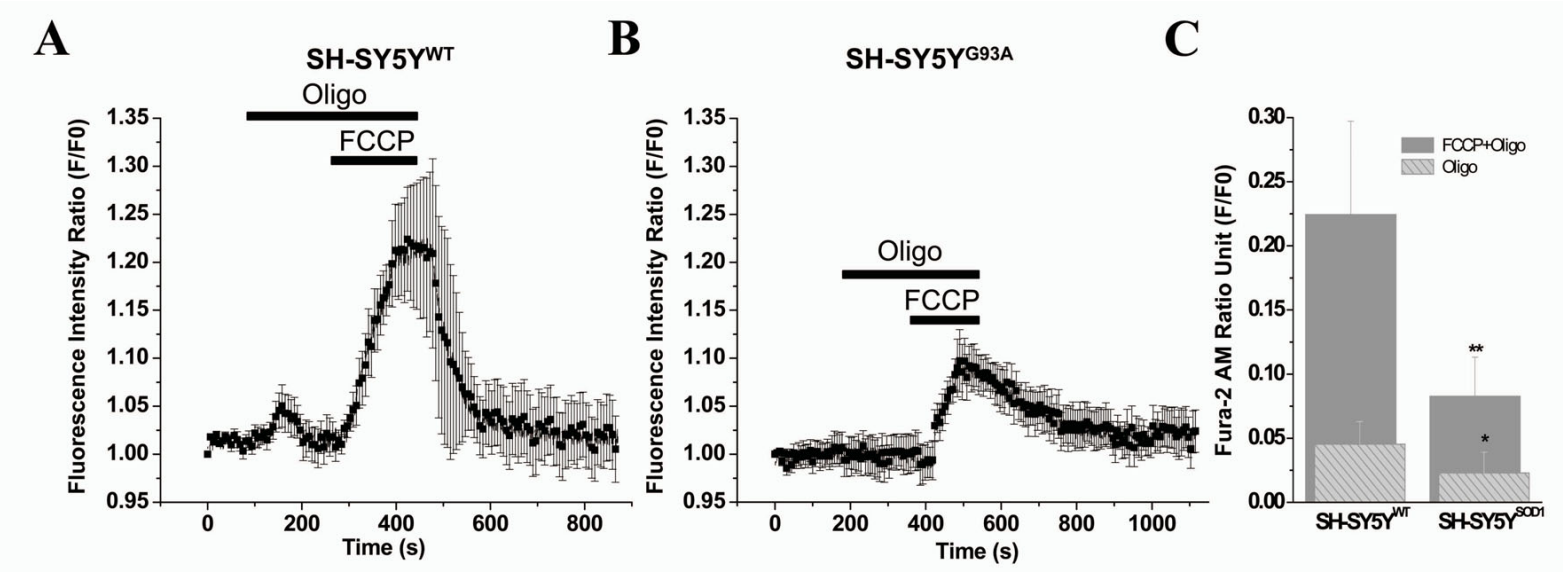
**Impact of inhibition of F1, F0-ATP synthase on FCCP-evoked responses of [Ca^2+^]i in  WT and G93A transfected SH-SY5Y cells**. A) The quantitative kinetic profile of the oligo and FCCP-evoked [Ca^2+^]i release in WT transfected SH-SY5Y cells. B) The corresponding quantitative kinetic profile of the oligo and FCCP-evoked [Ca^2+^]i release in the G93A transfected SH-SY5Y cells. The trace is representative of mean of 4-6 cells in focus stimulated with oligo (5 μg/ml; 6 min) and 2µM FCCP (3 min, normalized data). The horizontal black bars indicate the duration of stimulation by oligo and with FCCP plus oligo. Fura-2 AM signals are represented as F/F0. C) A bar diagram of oligo and FCCP plus oligo-induced Ca^2+^ release in the WT (N=3, n=20) and G93A (N=3, n=19) transfected SH-SY5Y neuroblastoma cells. Gray bars represent oligo plus FCCP-induced Ca^2+^ release in WT (F/F0 = 0.2245 ± 0.0727) and G93A (F/F0 = 0.0827 ± 0.0304) transfected SH-SY5Y cells. Striped bars represent oligo-induced Ca^2+^ release in WT (F/F0 = 0.0454 ± 0.0175) and G93A (F/F0 = 0.0229 ± 0.0161) transfected cells. Values represent means ± SD, *p<0.01, **p<0.001. N= Number of experiments; n= Number of cells.

As shown in Fig. 4, caffeine led to a relatively slow and weak increase in the [Ca^2+^]i fluorescence signals, which was very similar for both WT and G93A transfected cells (N = 3, n = 14). We found that WT transfected cells (Figs. 4A and 4C) were affected by 5 mM caffeine with a small and slow increase in [Ca^2+^]i (F/F0 = 0.0471 ± 0.0190) and exhibited slightly higher kinetics than the G93A transfected cells ([Ca^2+^]i: F/F0 = 0.0353 ± 0.0120; Figs. 4B and 4C). This suggests that the caffeine-evoked ER-dependent Ca^2+ ^release either makes only a minor contribution to mitochondrial mediated toxicity in G93A transfected cells, as reported in other cell types and animal models, or occurs upstream of the mitochondria.

The cells were treated with FCCP in the presence of caffeine, which resulted in the depolarization of the mitochondria (Fig. 4). We observed a rapid and high amplitude increase of [Ca^2+^]i, which exhibited a very distinct and differential response for WT and G93A transfected cells (N = 3, N = 14). WT transfected cells revealed that caffeine and FCCP together evoked a massive and comparatively fast increase in the Fura-2 fluorescence signals for WT (F/F0 = 0.1883 ± 0.0584, Figs. 4A and 4C) and G93A transfected cells (F/F0 = 0.1154 ± 0.0246, Figs. 4B and 4C).

### Manipulation of mitochondria in WT and G93A transfected SH-SY5Y cells by pharmacological Inhibition of F_1_, F_0_-ATP Synthase

As an uncoupler of mitochondria, FCCP collapses the mitochondrial membrane potential. In turn, this results in a rapid release of calcium from this store and a slower drop in ATP levels [64]. Impairment of the mitochondria with FCCP may block oxidative phosphorylation and has the potential to compromise oxidative phosphorylation; in this state, glycolysis provides the prime means of ATP synthesis. Previously, evidence had suggested that ATP depletion by FCCP in MNs leads to minimal effect on reverse cycle of uncoupling of oxidative phosphorylation ([62], S. Balakrishnan and B.U. Keller, unpublish data). We assume that the sole effect of FCCP on cells is the uncoupling of mitochondria and that this results might lead to a reduction of the proton gradient across the internal mitochondrial membrane. Oligo, an ATP synthase inhibitor, also depletes ATP but does not alter mitochondrial membrane potential and thus does not change calcium transport across the mitochondrial membrane. Oligo was added with FCCP to prevent any accelerated consumption of cellular ATP by the reverse mode of ATP synthase operation [65,66].

**Figure 5:**
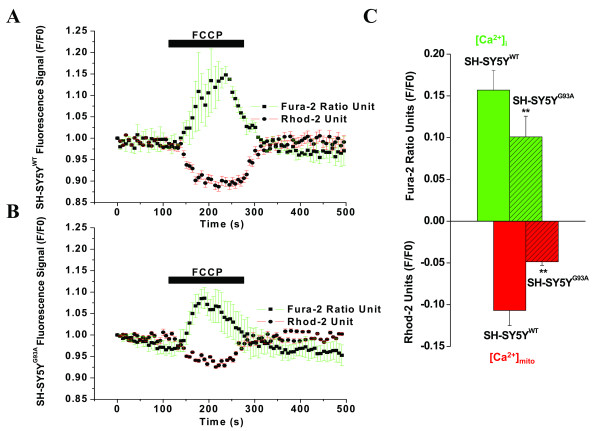
**The simultaneous measurement of cytosolic (Fura-2) and mitochondrial (Rhod-2) calcium concentrations in WT and G93A transfected transfected SH-SY5Y cells during FCCP-evoked mitochondrial Ca^2+^ release**. A) The kinetic profile of the FCCP-evoked Ca^2+^ release in the WT transfected SH-SY5Y neuroblastoma cells; the cytosolic (Error bar green, black square trace) and mitochondrial (Error bar red, black circle trace) compartment were measured simultaneously. The trace represents the mean of 5 cells in focus stimulated with 2µM FCCP (5 point smoothing).  B) The corresponding kinetic profile of the FCCP-evoked Ca^2+^ release in the G93A transfected SH-SY5Y neuroblastoma cells; the cytosolic (Error bar green, black square trace) and mitochondrial (Error bar red, black circle trace) compartment were measured simultaneously. The trace represents the mean of 5 cells in focus stimulated with 2µM FCCP (5 point smoothing). FCCP-evoked [Ca^2+^]mito signals were smaller in amplitude and exhibited slower kinetics in G93A transfected SH-SY5Y cells compared to WT transfected cells and were altered from [Ca^2+^]i efflux. C) A bar diagram of the cytosolic (green bar) and mitochondrial (red bar) fluorescence signals (F/F0) from WT (F/F0 = 0.1569 ± 0.0235 for [Ca^2+^]i and F/F0 = -0.1069 ± 0.0181 for [Ca^2+^]mito; hollow; N=5,  n=17) and G93A (F/F0 = 0.1008 ± 0.0248 for [Ca^2+^]i and F/F0 = -0.0486 ± 0.0043 for[Ca^2+^]mito; striped pattern, N=4; n=17) transfected SH-SY5Y neuroblastoma cells. Values represent means ± SD, **p<0.001. N= Number of experiments; n= Number of cells.

To clarify this issue, we measure the impact of oligo (5 μg/ml) on FCCP-induced Ca^2+ ^release in WT and G93A transfected SH-SY5Y cells loaded with fura-2. Very weak effects to those previously observed with the electron transport inhibitors (CN^-^) and azide were seen with oligomycin [19,62]. As shown in Figs. 5A–C, impact of oligo's on the normalized peak fluorescence of fura-2 was 0.0454 ± 0.0175 and 0.0229 ± 0.0161 for WT and G93A transfected SH-SY5Y cells, respectively (Fig. 5C). The impact of oligomycin on the peak amplitude of fura-2 fluorescence was nominal and there was slight difference between WT and G93A transfected SH-SY5Y cells. However, the response to FCCP plus oligomycin in WT transfected cells (Fig. 5C) was almost 2.71-fold higher (F/F0 = 0.2245 ± 0.0727) compared with G93A transfected SH-SY5Y cells (F/F0 = 0.0827 ± 0.0304; Figs. 5A–C).

### Simultaneous measurement of [Ca^2+^]i and [Ca^2+^]mito

**Figure 6:**
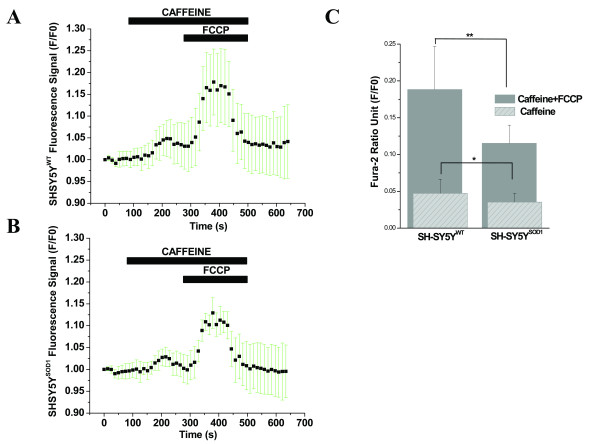
**Caffeine stimulates [Ca^2+^]i release with slower kinetics and weaker transient than the FCCP-evoked [Ca^2+^]i signals in SH-SY5Y neuroblastoma cells, particularly in WT transfected cells compared to G93A transfected cells**. A) The kinetic profile of caffeine and FCCP-evoked [Ca^2+^]i release in the WT transfected SH-SY5Y neuroblastoma cells. The trace is representative of 5 cells in focus stimulated with 5mM caffeine and 2µM FCCP (normalized data, 5 point smoothing). B) The corresponding kinetic profile of the caffeine and FCCP-evoked [Ca^2+^]i release in the G93A transfected SH-SY5Y cells. The trace is representative of 5 cells in focus stimulated with 5mM caffeine and 2µM FCCP (normalized data, 5 point smoothing). The ER and mitochondrial Ca^2+^ release from these two compartments were measured simultaneously. The horizontal black bars indicate the duration of stimulation by caffeine and with FCCP plus caffeine. Fura-2 AM signals are represented as F/F0.  C) A bar diagram of caffeine and FCCP plus caffeine-induced Ca^2+^ release in the WT and G93A transfected SH-SY5Y neuroblastoma cells (N=3, n=14). Gray bars represent caffeine plus FCCP-induced Ca^2+^ release in WT (F/F0 = 0.1883 ± 0.0584) and G93A (F/F0 = 0.1154 ± 0.0246) transfected SH-SY5Y cells. Striped bars represent caffeine-induced Ca^2+^ release in WT (F/F0 = 0.0471 ± 0.0190) and G93A (F/F0 = 0.0353 ± 0.0120) transfected cells. Values represent means ± SD, *p<0.01, **p<0.001. N= Number of experiments; n= Number of cells.

Using FCCP to prevent a [Ca^2+^]mito increase while measuring an increase in [Ca^2+^]i, we were able to efficiently separate cytosolic and mitochondrial Ca^2+ ^at a temporal resolution in the millisecond time domain. As shown in Fig. 1, the Ca^2+ ^release in G93A transfected cells is smaller in amplitude and exhibits slower kinetics compared to WT transfected cells. To confirm that there are separate Ca^2+ ^uptake and release events from the cytosol and mitochondria, the transfected cells were treated with FCCP and the separate Ca^2+ ^levels measured simultaneously (Fig. 6). WT transfected cells exhibited a substantial increase in [Ca^2+^]i (F/F0 = 0.1569 ± 0.0235; Fig. 6A, N = 5; n = 17) while [Ca^2+^]mito decreased (F/F0 = -0.1069 ± 0.0181, Fig. 6A, N = 5; n = 17). This suggests that the FCCP-evoked increase in [Ca^2+^]i in Fig. 1 was mainly due to [Ca^2+^]i and that there was very little impact from [Ca^2+^]mito. In G93A transfected cells, FCCP-evoked [Ca^2+^]i was less and exhibited slower kinetics (F/F0 = 0.1008 ± 0.0248; Figs. 6B and 6C, N = 4; n = 17) compared to WT transfected cells, which exhibited a reduction of the [Ca^2+^]mito (F/F0 = -0.0486 ± 0.0043, Figs. 6B and 6C, N = 4; n = 17).

## Discussion

Recent evidence suggests that abnormalities in cellular Ca^2+ ^signaling are common features in the pathogenesis of a range of neurodegenerative disorders, including ALS [33]. It is well known that Ca^2+ ^is one of the most relevant intracellular messengers essential in neuronal development, synaptic transmission and plasticity, and the regulation of various metabolic pathways in the brain. Further evidence for the involvement of a disruption in intracellular Ca^2+ ^homeostasis was reported in cellular and experimental animal models; there was an absence of Ca^2+ ^binding proteins, such as Calbindin-D_28K _and parvalbumin, in MN populations lost early in ALS [67]. Additionally, accumulation of Ca^2+ ^into vacuoles in the mtSOD1 mice and low Ca^2+ ^buffering in SMNs were also shown [68]. These findings agree with a quantitative comparison of Ca^2+^homeostasis where a low cytosolic Ca^2+ ^buffering capacity acts as an important risk factor for degeneration. In contrast, an increase in the cytosolic Ca^2+ ^buffering capacity could protect vulnerable MNs from degeneration [69,35]. MNs store a larger amount of calcium in the mitochondria and disruption of mitochondrial Ca^2+ ^uptake has a marked influence on both the peak amplitude of Ca^2+ ^response as well as the clearance of [Ca^2+^]i.

Our interest in studying the role of Ca^2+ ^regulation and the impact of mitochondrial inhibition in a cellular model of ALS is based on observations that mitochondria act as local calcium buffers, thus shaping the spatiotemporal aspects of [Ca^2+^]i. MN mitochondria have been shown to have a major percentage of Ca^2+ ^sequestered intracellularly after influx through the plasma membrane [70,71]. Our main objective was to characterize the contribution of the mitochondrial buffering of voltage-activated Ca^2+ ^loads and how this Ca^2+ ^regulatory mechanism is controlled. We observed that Ca^2+ ^influx was diminished by approximately 1.86 fold in the presence of G93A compared to WT (Fig. 1). After exposure to FCCP, the capacity of G93A transfected cells to transport [Ca^2+^]i to the extracellular space or to intracellular storage sites was much lower than WT transfected cells and accounted for the increased vulnerability of cells possessing mutant G93A gene.

Interestingly, the depolarization-induced Ca^2+ ^transient in the presence of intact mitochondria was smaller in amplitude than with G93A mitochondria; excess Ca^2+ ^emerged in the second response and was prominent in WT cells (Figs. 2A–C). This reflects the variability in Ca^2+ ^clearing mechanisms of ALS-vulnerable and non-vulnerable MNs and agrees with data obtained from motor nerve terminals [72], cultured MNs [25], and patch-clamped hypoglossal MNs [52] in which significant mitochondrial Ca^2+ ^uptake following voltage-activated Ca^2+ ^influx was demonstrated. We observed that the depolarization-induced stimulus not only interfered with the post-depolarization recovery of [Ca^2+^]i after FCCP challenge but also increased the peak amplitude of the FCCP-evoked Ca^2+ ^influx by approximately 40 and 68% in WT and G93A cells, respectively (compare Figs. 1D and 2C). Therefore, it is evident that in the absence of mitochondrial Ca^2+ ^uptake, the [Ca^2+^]i signal is stronger, and mitochondria can actively sequester Ca^2+ ^during an on-going Ca^2+ ^influx as a result of the opening of voltage-gated calcium channels. A significant difference in the Ca^2+ ^accumulation by mitochondria in G93A and WT cells was observed and presumably due to G93A-mediated toxicity. The capacity of mitochondria to restore the Ca^2+ ^buffering capacity of cell organelles is heavily compromised in G93A cells. The disturbance of Ca^2+ ^trapping in intracellular storage sites and Ca^2+ ^extrusion from the cells [41] may account for the reduced oscillations of [Ca^2+^]i we observed in G93A cells.

Molecular interaction underlying mitochondria-endoplasmic reticulum Ca^2+ ^strores coupling was evaluated using thapsi and FCCP. We assume that both ER and mitochondrial intracellular pools participate in the generation of Ca^2+ ^signals in SH-SY5Y cells shaping their spatiotemporal Ca^2+ ^signals patterns. Thapsi- induced Ca^2+^release was significantly less than that evoked by FCCP in WT and G93A transfected cells, and its kinetics were more or less similar in both genotypes (Figs. 3A–C). The application of thapsi on SH-SY5Y cells causing separate Ca^2+ ^release response was slightly more in WT transfected cells than in G93A cells, indicating that, in the context of the G93A transfection, the ER may slightly contribute to the motor dysfunction. Furthermore, the application of FCCP after emptying ER stores with thapsi resulted in a separate release event, evident from the bigger [Ca^2+^]i increase. This release was higher than the general Ca^2+ ^release caused by FCCP without emptying ER in WT transfected cells but not in G93A altered cells, which suggests an uptake of the released Ca^2+ ^from ER by mitochondria in WT transfected cells. This further indicates the explicit action of FCCP in our working model system and the existence of two separate intracellular Ca^2+ ^stores, in which the ER seems to play a minimal role in buffering [Ca^2+^]i after Ca^2+ ^loads are imposed in G93A transfected cells, and the ER is most likely not impaired during ALS-related motoneuron disease.

We found that ER of SH-SY5Y cells retained a very low amount of calcium (after Ca^2+ ^release with thapsi for 6 min application) compared with mitochondria after Ca^2+ ^elevation by FCCP (3 min) indicating its low efficiency to sequester Ca^2+ ^in the WT and G93A transfected SH-SY5Y cells, which was slightly higher in WT (Figs. 3A–C). This indicates that the conventional mitochondrial Ca^2+ ^storing function dominates ER Ca^2+^accumulation in these cells. It is noteworthy that thapsi has a relatively very weak effect on Ca^2+ ^release in G93A transfected cells, indicating that G93A alteration might also result in defects in ER Ca^2+ ^handling, which may perturb functional domain and contribute to neurodegeneration. These data suggest that the ER of SH-SY5Y cells does not play a significant role in regulating [Ca^2+^]i at the basal level or after imposed Ca^2+^loads in WT transfected cells; though minor ER contribution to the dysfunction of Ca^2+ ^loads did not ruled out, suggesting that Ca^2+^dysregulation as a result of the G93A transfection anticipates mitochondrial impairment. Our hypothesis is strengthened by the fact that previously we have shown quite similar results in an animal model of SOD1^G93A ^mice for fALS; where cyclopiazonic acid (a specific inhibitor of Ca^2+^-ATPase led to a relatively slow and weak increase of [Ca^2+^]i, which occurred with slightly higher kinetics in WT HMNs than the SOD1^G93A ^mice HMNs [62]. This finding alongwith our results in WT and G93A transfected cells further explains that ER-dependent Ca^2+^release is a minor contributor to mitochondria-mediated toxicity in G93A transfected cells as previously reported in other cell types and animal models [73,74]. Results indicate close coupling between ER and mitochondria in WT but not in G93A transfected cells which is probably impaired due to G93A transfection. The thapsi-releasable Ca^2+ ^pool in WT and G93A transfected cells (Figs 3A–C) was quite similar in quantity and temporal quality release via RyR-sensitive Ca^2+ ^pool by caffeine (see Figs 4A–C). This observations suggests that Ca^2+ ^uptake by RyR-responsive pool is also sensitive to thapsi. These findings also indicate that thapsi increases [Ca^2+^]i by inhibiting Ca^2+ ^uptake into multiple intracellular Ca^2+ ^pools and by also promoting entry of extracellular Ca^2+ ^dominantly in WT but not in G93A transfected cells. The exact molecular interactions defining the organization of mitochondria, ER and other Ca^2+ ^sources varies among different cell types, are questions that remain unanswered but are interesting areas for future investigations.

We expect a decrease in cellular ATP levels during inhibition of mitochondrial electron transport by FCCP and this may affect release of [Ca^2+^]i. To test the impact of ATP depletion on regulation of [Ca^2+^]i, oligo (5 μg/ml) was added before FCCP application. Oligo does not influence [Ca^2+^]i heavily and left basal Ca^2+ ^levels, as well as recovery times of Ca^2+ ^transients, unaffected for upto several minutes (6 min application; Figs 5A–C) and therefore amplitude of evoke [Ca^2+^]i transients was clearly attributable to FCCP. Since application of oligo alone did not affect the [Ca^2+^]i transients therefore the effect of FCCP is clearly attributable to collapse of mitochondria, and not to inhibition of ATP-synthesis. We assume that the time course of ATP depletion by FCCP is slower but the increase of cytosolic calcium by FCCP is immediate and therefore there is no causal relationships have been demonstrated between fall in ATP level and increase of [Ca^2+^]i. These results are in agreement with those from studies of SOD1^G93A ^mice in sensitivity of HMNs mitochondria to oligo [62].

Using the CCD camera imaging system, we clearly separated the dynamic profiles of [Ca^2+^]i and [Ca^2+^]mito. Using this method, we demonstrated that in WT cells, the FCCP-evoked increase in [Ca^2+^]i was mainly due to [Ca^2+^]i alone. However, in G93A cells, the FCCP-evoked [Ca^2+^]i was less, exhibited slower kinetics, and reduced [Ca^2+^]mito. This suggests that cells with G93A have a lower affinity for mitochondrial Ca^2+ ^upload and likely represents severely disturbed and vulnerable mitochondria. In this study, for the first time, the simultaneous determination of [Ca^2+^]i and [Ca^2+^]mito in WT and G93A transfected SH-SY5Y cells, a culture model of motor neuron disease was achieved (Figs 6A–C). This data is in agreement with previous findings that G93A cells exhibit damaged and stressed mitochondria [6] and increased ROS [47].

Different intracellular pools participate in the generation of neuronal Ca^2+ ^signals, shaping their spatio-temporal patterns, and the cell life-death cycle. Mitochondria, in the kinetic and hot spot hypothesis, and the ER, by different classes of channels with distinct properties and highly defined expression patterns, have been implicated in the regulation of [Ca^2+^]_i _in many systems [41,42]. Caffeine was used to investigate ER-dependent Ca^2+ ^release and we found that it evoked a small and slow increase in [Ca^2+^]i with slightly faster kinetics in WT cells than G93A cells. This further suggests that ER-dependent Ca^2+ ^release has a minor contribution in the mitochondria-mediated toxicity of G93A. The rate of increase in Fura-2 fluorescence following caffeine application was slower and did not resume the baseline, which is likely due to the slow activity of the mitochondrial Na^+^/Ca^2+ ^exchanger, the major pathway for mitochondrial Ca^2+ ^efflux [75]. Observations of different pharmacological conditions support the concept that the presence of G93A severely disrupts mitochondrial Ca^2+ ^regulation. It is also interesting to note that the rate of increase in Fura-2 fluorescence signals following caffeine application was slightly slower compared to FCCP-evoked signals and did not achieve the baseline within a few minutes. This suggests that the dye accumulates in the mitochondria and the mitochondrial uptake and release event is prominently operated by Ca^2+ ^efflux rather than by the store-operated Ca^2+ ^uptake and release phenomenon. Our fluorescence system may be a valuable tool to determine mitochondrial and Ca^2+^-related defects during G93A-mediated MN degeneration, which closely parallels the incidence of neuronal death in G93A transfected SH-SY5Y cells. Quantitative values of normalized Fura-2 fluorescence signals after different drugs interventions are compared in Table 1.

**Table 1:** Differential peak amplitudes of [Ca^2+^]i release from different compartment of WT and SOD1 transfected SH-SY5Y cells after multidrugs intervention.

	FCCP	K+FCCP	Thapsi	Thapsi+FCCP	Oligo	Oligo+FCCP	Caffeine	Caffeine+FCCP
WT	0.17 ± 0.03	0.24 ± 0.06	0.04 ± 0.01	0.27 ± 0.09	0.04 ± 0.01	0.22 ± 0.07	0.04 ± 0.01	0.18 ± 0.05
SOD1	0.09 ± 0.02**	0.14 ± 0.03**	0.02 ± 0.01*	0.12 ± 0.02**	0.02 ± 0.01*	0.08 ± 0.03**	0.03 ± 0.01*	0.11 ± 0.02**

Data are expressed as mean ± S.D.

* *p *< 0.01 and ** *p *< 0.001 compared with WT transfected SH-SY5Y cells.

Despite rigorous research since Charcot's description more than 130 years ago, the molecular abnormalities leading to the damage of specific MNs in ALS are still unknown. The selective vulnerability of MNs in ALS-related disease and associated cell culture models is closely linked to exceptional Ca^2+ ^signaling mechanisms that are part of the physiological cell function, but seemingly also enhances the risk of Ca^2+ ^homeostasis disruption and mitochondrial dysfunction in vulnerable cells. Earlier studies suggested that uncontrolled Ca^2+ ^entry and inefficient calcium sequestering cause selective damage leading to the formation of vacuoles derived from the degenerating mitochondria in the MNs of the mouse model of ALS [12,76,77]. In contrast to most other neurons, MNs have a low Ca^2+^-buffering capacity due to the low expression of Ca^2+^-buffering proteins and a high number of Ca^2+^-permeable AMPA receptors resulting from low expression of the GluR2 subunit. The combination of these two properties seems to be intrinsic to MNs and is most likely essential for their normal function. However, under pathological conditions, MNs may become over-stimulated by glutamate and overwhelmed by Ca^2+^; though, whether downstream pathways activated by the intracellular Ca^2+ ^increase are different in MNs compared to other neurons is not yet known.

## Conclusion

**Figure 7:**
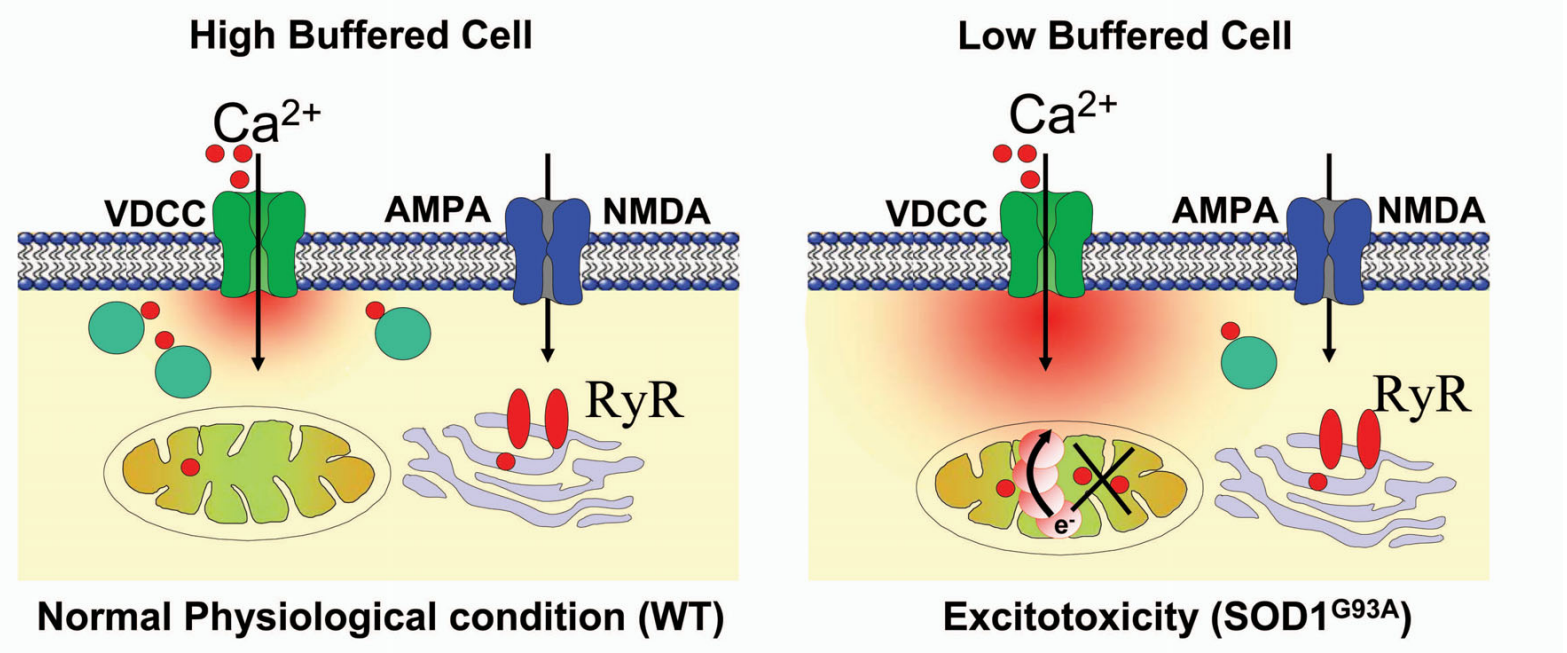
**Low Ca^2+ ^buffering and excitotoxicity under physiological stress and pathophysiological conditions in motor neuron (MNs)**. Low Ca^2+ ^buffering in amyotrophic lateral sclerosis (ALS) vulnerable hypoglossal MNs exposes mitochondria to higher Ca^2+ ^loads compared to highly buffered cells. Under normal physiological conditions, the neurotransmitter opens glutamate, NMDA and AMPA receptor channels, and voltage dependent Ca^2+ ^channels (VDCC) with high glutamate release, which is taken up again by EAAT1 and EAAT2. This results in a small rise in intracellular calcium that can be buffered in the cell. In ALS, a disorder in the glutamate receptor channels leads to high calcium conductivity, resulting in high Ca^2+ ^loads and increased risk for mitochondrial damage. This triggers the mitochondrial production of reactive oxygen species (ROS), which then inhibit glial EAAT2 function. This leads to further increases in the glutamate concentration at the synapse and further rises in postsynaptic calcium levels, contributing to the selective vulnerability of MNs in ALS.

Our experiments identified specialized Ca^2+ ^homeostasis characterized by low cytosolic Ca^2+ ^buffering in which mitochondria play a major role in the regulation of [Ca^2+^]_i _transients in vulnerable MNs. Low cytosolic Ca^2+ ^buffering enhances the role of low affinity organelle buffers, such as mitochondria, in the cell. For example, large and long-lasting Ca^2+ ^domains around influx sites enhance the risk of toxic Ca^2+ ^accumulations and subsequent activation of Ca^2+^-dependent neurodegenerative pathways under excitotoxic conditions. Indeed, a strong contribution of mitochondria, as opposed to ER, Ca^2+ ^uptake to the buffering of Ca^2+ ^profiles was recently demonstrated [19,52,62]. There are two reasons that the prominent role of mitochondria in the regulation of moderate Ca^2+ ^loads in MNs has important implications for pathological conditions such as ALS. First, the amount of Ca^2+ ^taken up by the mitochondria is probably higher in MNs than other cell types [25,78]. These high Ca^2+ ^loads enhance the risk for ROS generation, which may play a major role in initiating the death cycle resulting in MN degeneration [29]. Second, our experiments provide evidence that cytosolic Ca^2+ ^depends on intact mitochondrial Ca^2+ ^uptake. Thus, when mitochondrial Ca^2+ ^uptake is disturbed, as was seen in G93A cells, MNs are directly put at risk of accumulating basal Ca^2+ ^levels during repetitive oscillations. Additionally, the decreased ability to limit Ca^2+ ^transient amplitudes in the cytosol and, in particular, local domains when mitochondria are depolarized enhances the risk of initiating Ca^2+ ^dependent neurodegenerative pathways leading to cell death. A summary of the identified mechanisms is given in Fig. 7; in vulnerable MNs, the calcium buffering machinery is represented by the predominance of mitochondria and calcium binding proteins.

## Abbreviations

ALS: amyotrophic lateral sclerosis; AM: acetoxy methyl ester; CCD: charge cooled device; DMEM: Dulbecco's modified Eagle medium; DMSO: dimethyl sulfoxide; ER: endoplasmic reticulum; F: fluorescence; fALS: familial amyotrophic lateral sclerosis; FCCP: Carbonyl cyanide p-(trifluoromethoxy) phenylhydrazone; FCS: fetal calf serum; FMNs: facial motoneurons; HMNs: hypoglossal motoneurons; hALS: human amyotrophic lateral sclerosis; MCU: mitochondrial calcium uniporter; MN: motoneuron; mtSOD1: mutant Cu/Zn superoxide dismutase1; oligo: oligomycin; ROI: regions of interest; ROS: reactive oxygen species; RPMI 1640: Roswell Park Memorial Institute medium; RyR: ryanodine receptor; SD: standard deviation; SOD1: Cu/Zn superoxide dismutase1; TPP^+^: Tetraphenylphosphonium salt; thapsi: thapsigargin; [Ca^2+^]i: cytosolic calcium; [Ca^2+^]mito: mitochondrial calcium; WT: wild type; Δψ_m_: mitochondrial membrane potential.

## Competing interests

The authors declare that they have no competing interests.

## Authors' contributions

MKJ performed the experiments, analyzed the data, and wrote the initial version of the manuscript. WDZ, MG and RN established the primary cultured human SH-SH5Y cell line and maintained them during the course of the project. Cell lines expressing either wild-type (Wt) human SOD1 or the G93A mutants of this enzyme were produced by AF and MTC. CL, AZ, MTC and RN participated in the design of the study and participated in the organization of financial support. BUK is the principal investigator of the project. Besides providing laboratory support and supervision, he is primarily responsible for financial support and project management. All authors read and approved the final manuscript.

## Supplementary Material

Additional file 1 **Schematic representation of method and spectra**. Schematic representation of the (A) CCD-imaging setup used for calcium imaging and (B) simultaneous measurements of [Ca^2+^]i and [Ca^2+^]mito in SH-SY5Y cell line preparations. C) Spectral view of calcium imaging. The fluorescence excitation at 510 nm and emission detected at 340 nm are shown for Ca^2+^-saturated (A) and Ca^2+^-free (B) Fura-2 in pH 7.2 buffer. D) Spectral view of simultaneous [Ca^2+^]i and [Ca^2+^]mito measurements generated by the Molecular Probes spectral view program. Fluorescence excitation of fura-2 was done at 390 nm and 550 nm of rhod-2, respectively; emission was at 510 nm (fura-2, solid line) and at 600 nm (rhod-2) separated by a 565 nm dichroic mirror. Click here for file

Additional file 2 **Mitochondria-dependent responses of cytosolic calcium in SH-SY5Y neuroblastoma cells (non-transfected parental cell line) loaded with Fura-2 AM and superfused with DMEM medium**. A) Representative CCD camera photomicrograph of Fura-2 AM loaded SH-SY5Y cells. Scale bar is 10 μm. B) FCCP-evoked mitochondria-dependent responses of cytosolic calcium. Click here for file
