# Supplementary figures and images for: Impairment of mitochondrial calcium handling in a mtSOD1 cell culture model of motoneuron disease
Source: BMC Neurosci. 2009 Jun 22;10:64. doi: 10.1186/1471-2202-10-64 (PMC2716351; doi:10.1186/1471-2202-10-64)

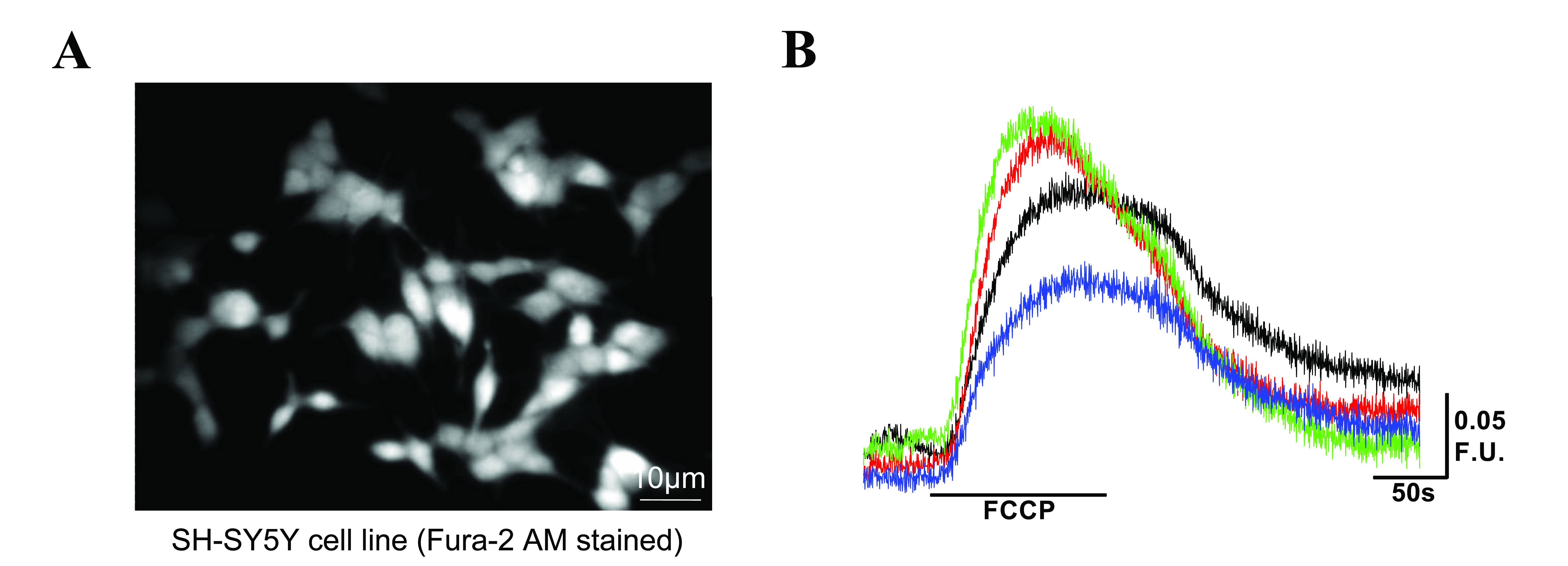

Supplement: Additional file 2 — Mitochondria-dependent responses of cytosolic calcium in SH-SY5Y neuroblastoma cells (non-transfected parental cell line) loaded with Fura-2 AM and superfused with DMEM medium. A) Representative CCD camera photomicrograph of Fura-2 AM loaded SH-SY5Y cells. Scale bar is 10 μm. B) FCCP-evoked mitochondria-dependent responses of cytosolic calcium. [file 1471-2202-10-64-S2.tiff]
